# Phasic alerting in visual search tasks

**DOI:** 10.3758/s13414-024-02844-3

**Published:** 2024-01-19

**Authors:** Niklas Dietze, Christian H. Poth

**Affiliations:** Department of Psychology, Neuro‑Cognitive Psychology and Center for Cognitive Interaction Technology, Bielefeld University, P.O. box 10 01 31, 33501 Bielefeld, Germany

**Keywords:** Phasic alertness, Visual search, Arousal

## Abstract

Many tasks require one to search for and find important objects in the visual environment. Visual search is strongly supported by cues indicating target objects to mechanisms of selective attention, which enable one to prioritise targets and ignore distractor objects. Besides selective attention, a major influence on performance across cognitive tasks is phasic alertness, a temporary increase of arousal induced by warning stimuli (alerting cues). Alerting cues provide no specific information on whose basis selective attention could be deployed, but have nevertheless been found to speed up perception and simple actions. It is still unclear, however, how alerting affects visual search. Therefore, in the present study, participants performed a visual search task with and without preceding visual alerting cues. Participants had to report the orientation of a target among several distractors. The target saliency was low in Experiment 1 and high in Experiment 2. In both experiments, we found that visual search was faster when a visual alerting cue was presented before the target display. Performance benefits occurred irrespective of how many distractors had been presented along with the target. Taken together, the findings reveal that visual alerting supports visual search independently of the complexity of the search process and the demands for selective attention.

How visual attention operates to support visual search is a matter of long-standing debate (Bundesen, 1990; Desimone & Duncan, 1995; Duncan & Humphreys, 1989; Treisman & Gelade, 1980; Wolfe, 1994, 2021). Serial models of attention in visual search assume that after the processing priorities of objects have been determined, there is a capacity-limited processing stage during which the objects are attended to one by one in sequence until the target object is found or detected (Treisman, 1988; Treisman & Gelade, 1980; Wolfe, 1994, 2021). In contrast, parallel and capacity-limited models assume that after the computation of object priorities, attention is distributed simultaneously across all objects in the visual scene according to their respective priorities (Bundesen, 1990; Desimone & Duncan, 1995). Both classes of models highlight the limited resources of the human brain, either the processing resources allocated to the objects (in parallel models) or the time needed to process each object (in serial models). It is still unclear, however, whether the available resources are constant over time or whether they fluctuate and depend on the state of the organism. It has been proposed that the amount of available resources could depend on the current state of arousal in the brain (Bundesen et al., 2015; Petersen & Posner, 2012; Posner & Petersen, 1990) or, more specifically, the state of alertness (Petersen & Posner, 2012; Posner & Petersen, 1990). Generally, alertness refers to the brain’s readiness for processing to support perception and action (Posner & Petersen, 1990; Sturm & Willmes, 2001). Alertness can be increased for short periods by introducing warning stimuli, which is referred to as phasic alertness (Sturm & Willmes, 2001). Even though we may speculate that phasic alertness can increase the available resources for visual processing, it is unclear how it affects performance in visual search tasks.

In lab-based studies, phasic alertness is typically evoked by presenting alerting cues before visual targets. Under these alerting conditions, reaction times are shorter (Dietze & Poth, 2022; Fan et al., 2002; Poth, 2020), visual processing speed is higher (Matthias et al., 2010; Petersen et al., 2017; Wiegand et al., 2017), perceptual sensitivity is increased (Li et al., 2018), or response accuracy is traded for shorter reaction times (Han & Proctor, 2022; McCormick et al., 2019; Posner et al., 1973). These benefits provided by alerting cues are referred to as alerting effects (Posner & Petersen, 1990). It has been proposed that a distinct neural network including frontal and parietal areas of the human brain makes up the alerting system (Petersen & Posner, 2012). The alerting network is supposed to be functionally independent but interrelated to other subsystems of attention (Posner & Petersen, 1990). In particular, it has been found that alerting interacts with orienting of attention and overrides spatial deficits in neglect patients (Callejas et al., 2005; Chandrakumar et al., 2019; Festa-Martino et al., 2004; Finke et al., 2012; Fuentes & Campoy, 2008; Ishigami et al., 2016; Robertson et al., 1998). This suggests that alerting might also influence selective attention and expedite target selection among distractors.

Initial findings on phasic alertness influencing performance in visual search tasks come from studies investigating auditory (Asutay & Västfjäll, 2017) and visual (Müller-Oehring et al., 2013) alerting effects with conditions varying in search task complexity. The study by Asutay and Västfjäll (2017) found that auditory alerting with environmental sounds reduced reaction times in more complex displays with low-salient targets, but this effect did not occur with easier displays containing high-salient (“pop-out”) targets. It was also found that the environmental sounds inducing higher arousal caused greater alerting effects than those inducing lower arousal levels. Müller-Oehring et al. (2013) found that visual alerting had a more pronounced effect when the visual load was increased by adding more distractors along with the target. Both studies seem to contradict the arousal-biased competition hypothesis, which suggests that arousal should strengthen objects that already received prioritised processing (e.g., salient targets, but weaken objects with lower priority; Mather & Sutherland, 2011). Accordingly, the additional arousal boost from the alerting cues should have facilitated the processing of the salient targets. However, it is possible that the efficient search tasks used in the studies by Asutay and Västfjäll (2017) and Müller-Oehring et al. (2013) might have already pushed performance to ceiling, leaving little room for further improvement. As a result, the additional arousal induced by the alerting cue may not have had a noticeable impact on task performance. Therefore, other studies on visual search are warranted that investigate alerting with varying search complexities.

Crucially, these previous studies have one major drawback as participants either knew exactly when the target display was presented (i.e., at sound offset; Asutay & Västfjäll, 2017) or could anticipate the onset of the target display (i.e., cue–target onset asynchronies [CTOA] drawn from a uniform distribution, leading to increasing temporal expectation with increasing CTOA; Niemi & Näätänen, 1981; Müller-Oehring et al., 2013; Weinbach & Henik, 2012), which should have reduced temporal uncertainty and also caused reaction time benefits (Correa et al., 2004, 2005; Coull & Nobre, 1998). However, the study by Asutay and Västfjäll (2017) showed that higher levels of self-reported arousal were associated with the alerting effect, in line with the idea that the alerting effect was driven by the arousal state rather than temporal expectation. So, based on these findings, and the challenges of disentangling alerting, temporal orienting, and temporal expectation, it is still unclear how phasic alertness impacts on visual search performance.

A more recent study investigated visual alerting effects in detection and discrimination search tasks (Jankovic et al., 2022). In this study, participants either had to detect a pop-out item or discriminate a component from a pop-out item. Surprisingly, it was found that a preceding visual alerting cue sped up detection search but had no effects in the more complex discrimination search. Benefits in the complex visual search task were only found when the pop-out item occurred at the same location as the previous trial, essentially turning the task into a simple visual search task, because spatial attention was already directed at that location. As such, these findings are at odds with the previously outlined studies that found no or smaller effects with easier tasks (Asutay & Västfjäll, 2017; Müller-Oehring et al., 2013). However, the study was limited to a single set size at a time and only used pop-out targets that were easily detectable.

In summary, the evidence showing that phasic alertness benefits both simple and more difficult visual search tasks is mixed. Therefore, in the present study, we employed two visual search tasks with different target and distractor discriminability, as well as several set sizes, to investigate the influence of visual alerting on visual search performance under different levels of complexity. In addition, we varied the influence of temporal expectation by utilising a fixed CTOA of 500 ms in Experiment 1 and CTOAs of 300, 500, or 700 ms drawn from a geometric distribution in Experiment 2. The CTOAs were chosen based on previous work that has proposed the most optimal impact on performance with intervals of around 500 ms (Posner & Boies, 1971). That way, we were able to test the combined effects of phasic alertness and temporal expectation on visual search performance. We found that alerting resulted in similar reaction time benefits independent of the target saliency and number of distractors. This demonstrates that the mechanism of selective attention does not interact with phasic alertness in visual search processes. We also found that the reaction time benefits were greater with higher temporal certainty induced by the fixed CTOA.

## Experiment 1

### Method

In Experiment 1, participants had to respond to the orientation of a titled *T* among *T*-shaped distractors (low-saliency of targets). In 50% of trials, the target display was preceded by a visual alerting cue with a fixed CTOA of 500 ms. If phasic alertness affects visual search performance, we expect to observe shorter reaction times with a preceding alerting cue.

#### Participants

One hundred twenty-eight participants recruited via the participant pool of the Psychology Department at Bielefeld University took part in Experiment 1. Twenty-eight were males, 99 were females, and one identified as neither male nor female. They were between 16 and 59 years old (median = 23 years). Due to performance near chance level or incomplete data sets, 31 participants had to be excluded from the analyses, resulting in a final sample of 97 participants. The final sample consisted of 23 males, 73 females and one that identified as neither male nor female that were between 16 and 59 years old (median = 22 years). All participants reported normal or corrected-to-normal vision and confirmed a written consent before participation. The study was in accordance with the ethical guidelines of the German Psychological Association (DGPs) and approved by Bielefeld University’s ethics committee.

#### Apparatus and stimuli

The experiment was conducted online via Pavlovia (Open Science Tools Ltd., 2019) with the PsychoPy application (Pierce et al., 2019) that provides precise enough timing for reaction time experiments (Bridges et al., 2020; Dietze & Poth, 2022). To ensure consistent stimuli sizes across the participants’ monitors, we applied a credit card scaling procedure at the beginning of the experiment (Morys-Carter, 2021). In addition, participants were instructed to set their monitors to a refresh rate of 60 Hz, position themselves 65 cm away from the monitor, and use an external computer mouse placed in from of them for response collection. The refresh rate for all participants was obtained, revealing that all participants in Experiment 1 and 54 out of 60 in Experiment 2 set their refresh rate to 60 Hz. Stimuli were all white figures, presented on a black background. The white figures consisted of a fixation dot of approximately 0.2° of visual angle, a rectangle functioning as the alerting cue of approximately 9.8° of visual angle, and the distractors and target letter of approximately 1.4° of visual angle (given a 15.6-inch display at the instructed viewing distance). The locations from the distractors and target were approximately 3.2° of visual angle apart and randomly drawn from a 4 × 4 matrix.

#### Procedure

**Fig. 1:**
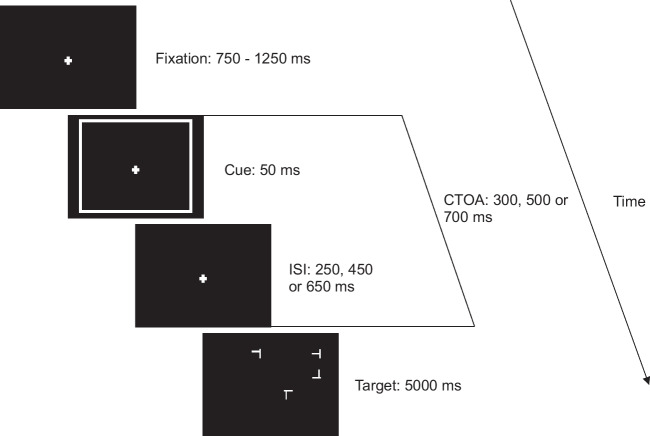
Trial procedure of Experiment 1 and Experiment 2. Participants fixated the centre of the screen and either no cue or a cue was presented, after which the target display appeared. In Experiment 1, the CTOA was fixed at 500 ms. In Experiment 2, the CTOA was either 300, 500, or 700 ms drawn from a geometric distribution

**Fig. 2:**
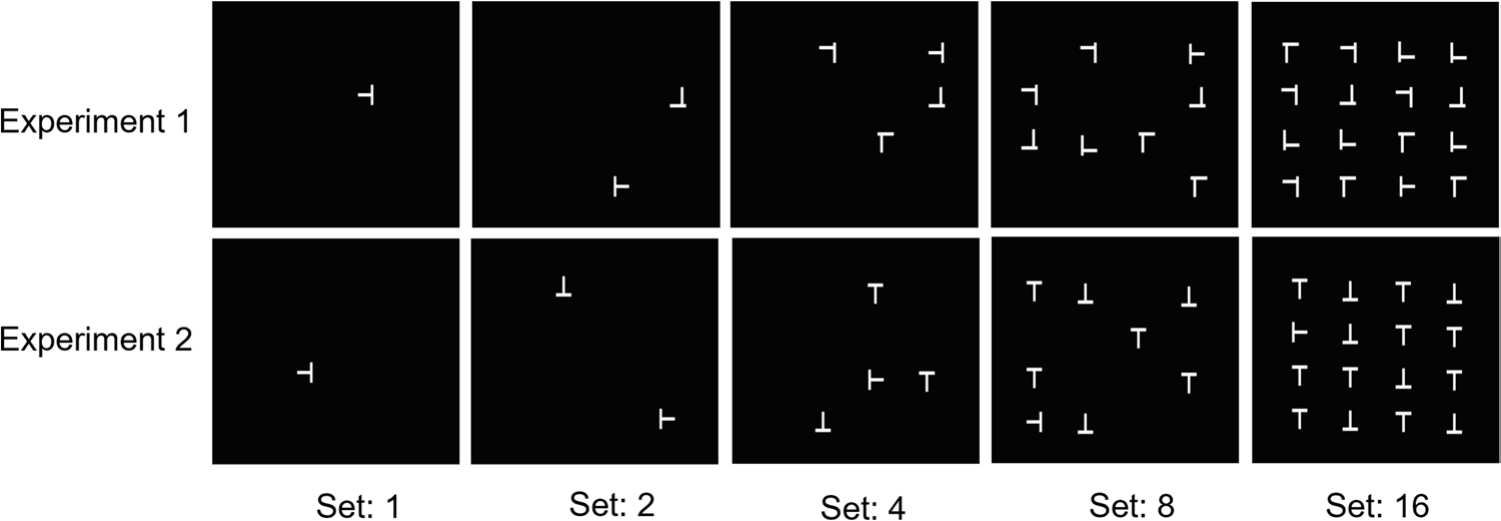
Target displays for different set sizes in Experiment 1 and Experiment 2

Trials started with the onset of the fixation dot. After a random stimulus interval of 750–1,250 ms drawn from a uniform distribution, either a cue (50% of trials) or no cue (50% of trials) was presented for 50 ms. The target display followed after a CTOA of 500 ms (see Fig. 1). The target display included either no distractor, one distractor, three distractors, seven distractors, or 15 distractors. Participants were asked to respond as quickly as possible to the orientation of a tilted *T*. They pressed the right mouse button if the target was tilted to the right relative to screen centre, and the left mouse button if the target was tilted to the left relative to screen centre (see Fig. 2). Each participant conducted five practice trials and 600 experimental trials presented in four blocks of 150 trials. The experiment lasted around 45 minutes. Reaction times and error feedback were only provided during the practice trials.

#### Analyses

Reaction times were compared in R (Version 4.1.2; R Core Team, 2021), with linear-mixed models including cue type (cue, no cue), set size (1, 2, 4, 8, 16), cue type × set size interaction and a random intercept by participant using a Gaussian distribution and an identity link function with the package lme4 (Version 1.1-27.1; Bates et al., 2015). Since the reaction times were positively skewed, we transformed the data by taking the negative inverse of the reaction times (cf. Brysbaert & Stevens, 2018). The linear mixed models are presented in Wilkinson notation as implemented in R (Wilkinson & Rogers, 1973). To check for sufficient power, we ran sensitivity analyses with power simulations based on alerting effects of 10, 15, and 20 ms using the package simr (Version 1.0.5; Green & MacLeod, 2016). Additionally, we tested for speed–accuracy trade-offs with logistic mixed models by investigating how accuracy was modulated by reaction times, cue type, set size, their interactions, and a random intercept by participant using a Binomial distribution and a logit link function with the same package lme4. We also arcsine square root transformed the accuracy rates and plotted conditional accuracy functions to visually aid the analyses (cf. Heitz, 2014). Practice trials, trials with anticipatory responses (reaction times < 100 ms) or without responses (2.8%) and trials with extremely long or short reaction times (reaction times > 2.5 *SD*s of the respective participant; 2.4%) were excluded. For the reaction time analyses, we additionally excluded error trials (7.4%).

#### Transparency and openness

All data sets and analysis scripts are available on the Open Science Framework (https://osf.io/w24zd/).

### Results and discussion

**Fig. 3:**
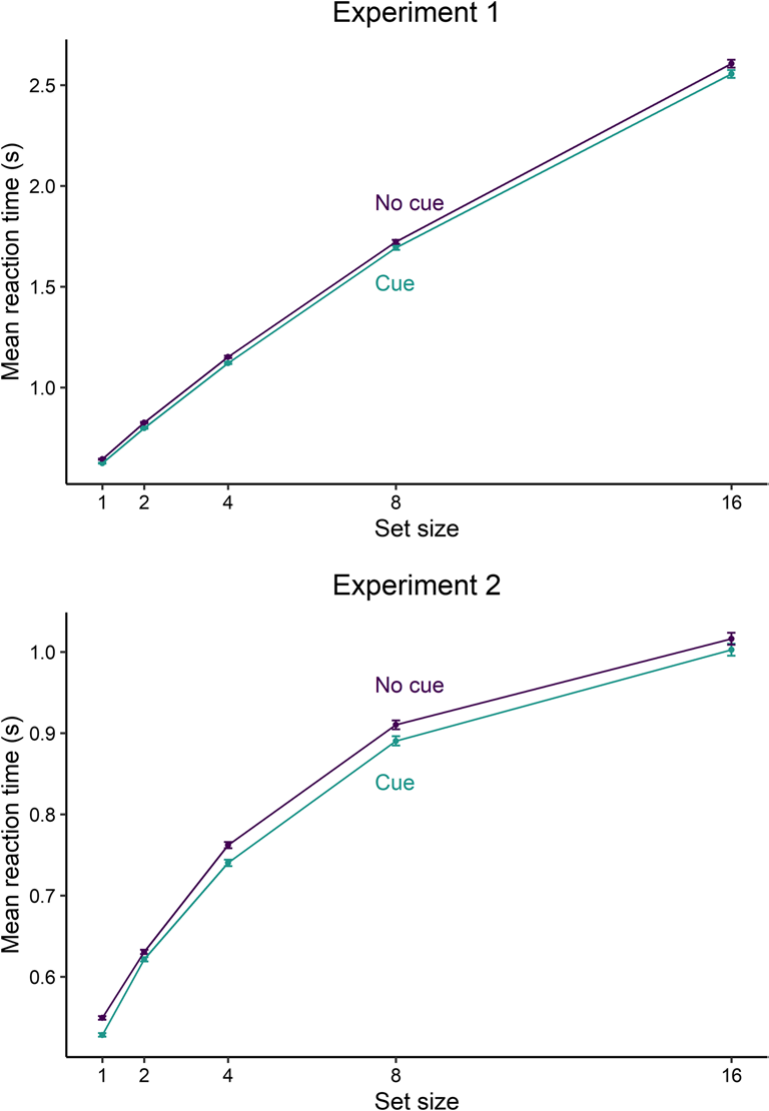
Mean reaction times in Experiment 1 and Experiment 2. Error bars depict the standard error of the mean

The linear mixed model (*inverse reaction times = cue × scale(set size) + (1 | participants)*) revealed a strong main effect of alerting (*β* = 0.036, *t* = 9.642, *p* < .001), showing shorter reaction times for trials with a preceding alerting cue than for trials without a cue. On average participants needed 1,353 ms (*SD* = 969 ms) in trials without a cue and 1,318 ms (*SD* = 958 ms) in trials with an alerting cue. Given alerting effects (reaction time difference between no cue trials and alert trials) of 10, 15, and 20 ms, the power simulations resulted in a power of 82% [73.05, 88.97], 96% [90.07, 98.90], and 100% [96.38, 100], respectively. We also found a main effect of set size (*β* = 0.365, *t* = 139.456, *p* < .001), showing faster reaction times for target displays with fewer distractors. Each additional distractor increased the reaction times by about 130 ms, showing that the search was difficult (see Fig. 3), which aligns with the characteristic set size effect in conjunction search tasks (Treisman & Sato, 1990). The interaction between cue and set size was also significant (*β* = −0.012, *t* = −3.278, *p* = .001).

**Fig. 4:**
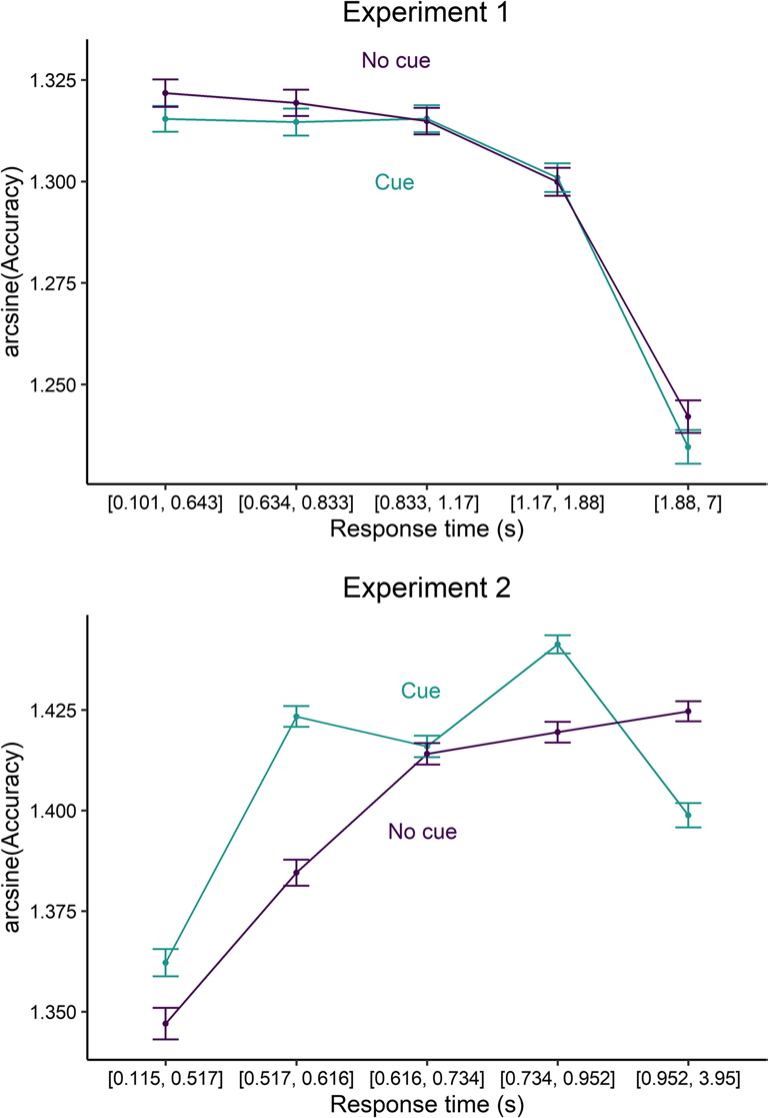
Conditional accuracy functions with arcsine square root transformed accuracy rates covering five equal-sized bins for Experiment 1 and Experiment 2. Error bars depict the standard error of the mean

Complementary to the reaction time analyses, we examined the accuracy rates with a logistic mixed model (*accuracy = reaction time × cue × scale(set size) + (1 | participants)*). We found that participants’ accuracy was not significantly influenced by cue type (*β* = 0.066, *z* = 0.807, *p* = .420), cue × reaction times interaction (*β* = −0.052, *z* = −0.788, *p* = .431) or cue × reaction times × set size interaction (*β* = 0.039, *z* = 1.030, *p* = .303), which rules out a speed–accuracy trade-off caused by the alerting cue (see Fig. 4). Here, we only found a significant interaction between reaction times and set size (*β* = −0.145, *z* = −5.314, *p* < .001), showing that the average accuracy was higher with faster reaction times and fewer distractors, but lower with faster reaction times and more distractors.

**Fig. 5:**
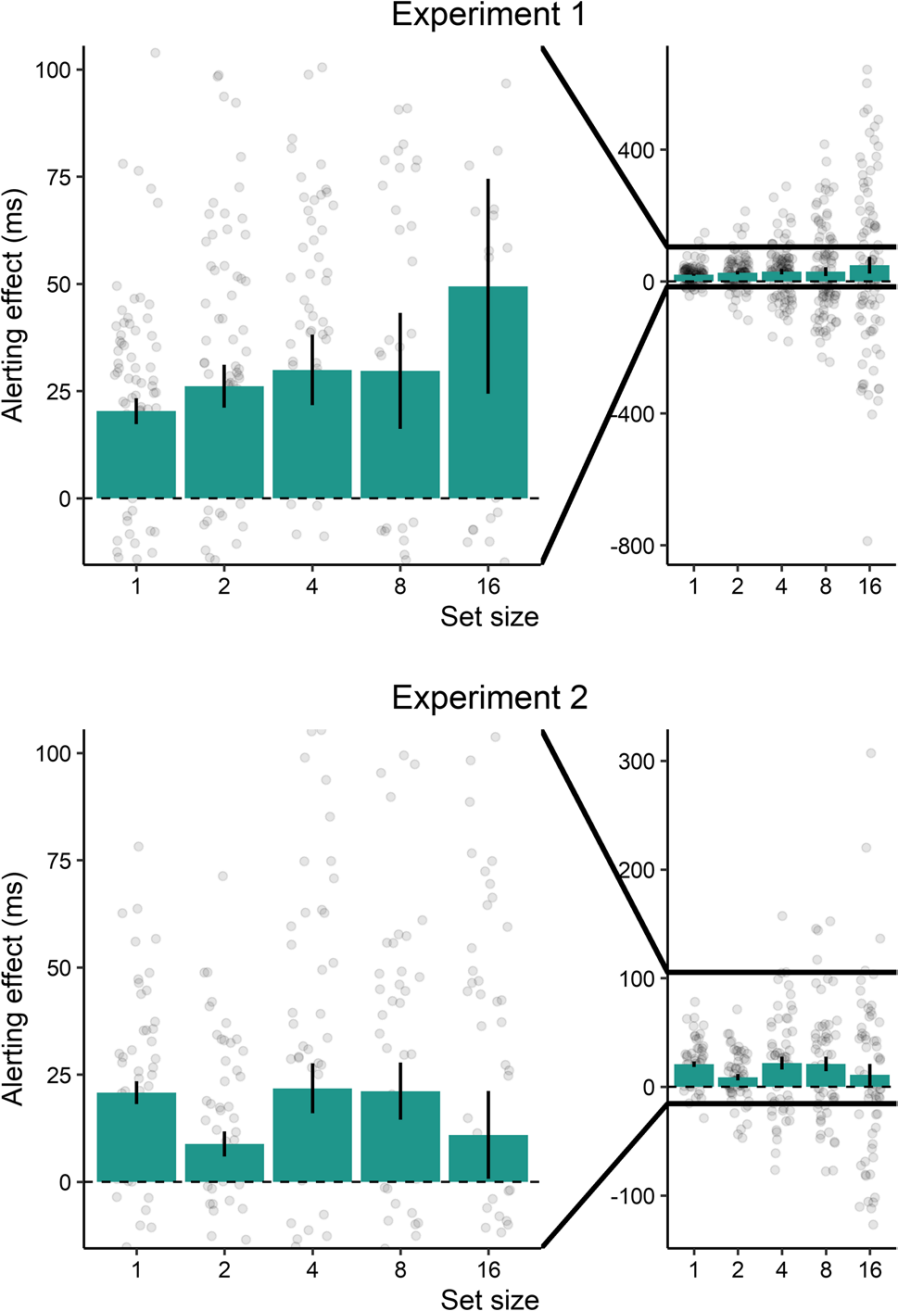
Alerting effects (reaction time difference between no cue trials and alert trials) in Experiment 1 and Experiment 2. The left part of the plot is the magnified version of the overall results presented on the right. Error bars depict the standard error of the mean. Transparent points represent the individual alerting effects

The results of Experiment 1 show that visual alerting benefits occurred in a visual search task with low target and distractor discriminability (low-saliency of targets). Although the interaction reached significance, we found that the alerting effects were relatively similar across all set size conditions except for the condition with 16 stimuli (see Fig. 5). Here, the variability was greatest. Yet these findings could potentially be driven by the constant CTOA, so that participants always knew when to expect the onset of the search display. Notably, the condition with 16 stimuli appeared to derive the greatest benefit from temporal expectation, likely due to its higher complexity, leaving more room for improvement. However, it is crucial to note that these observations do not imply a direct link between temporal expectation and set size. Instead, the findings suggest that more difficult conditions are more prone to experiencing beneficial effects, as participants are generally further from reaching a performance ceiling.

## Experiment 2

In Experiment 2, we increased temporal uncertainty by using CTOAs of 300, 500, or 700 ms drawn from a geometric distribution (Petersen et al., 2017; Poth, 2020; Poth et al., 2014; Weinbach & Henik, 2012). In addition, we reduced the difficulty of the visual search task by changing the distractor properties because previous research has shown that alerting effects are smaller or absent when the search process is more efficient (Asutay & Västfjäll, 2017; Müller-Oehring et al., 2013). Therefore, in Experiment 2, we used distractors that only consisted of the letter *T* with a different orientation than the target letter. This modification made it easier to distinguish the target from the distractors (high saliency of targets). While the visual search task in Experiment 2 may still be considered challenging, the various distractor conditions provide insights into searches that are relatively easy versus those that are more difficult.

### Participants

Eighty-three participants recruited from the same participant pool performed Experiment 2. Twenty-seven were males, 54 were females, and two identified as neither male nor female. They were between 17 and 42 years old (median = 22 years). Due to performance near chance level or incomplete data sets, 23 participants had to be excluded from the analyses, resulting in a final sample of 60 participants. The final sample consisted of 23 males, 35 females, and two that identified as neither male nor female who were between 17 and 42 years old (median = 22 years).

#### Apparatus and stimuli

The apparatus and stimuli were identical to that of Experiment 1.

#### Procedure

The procedure was the same as Experiment 1, except for the following. The target display followed after a CTOA of 300, 500, or 700 ms drawn from a geometric distribution with a hazard rate of 0.5. Each participant conducted five practice trials and 560 experimental trials presented in four blocks of 140 trials. The experiment lasted around 40 minutes.

#### Analyses

The statistical analyses followed those of Experiment 1. Practice trials, trials with anticipatory responses (reaction times <100 ms) or without responses (0.1%) and trials with extremely long or short reaction times (reaction times >2.5 *SD*s of the respective participant; 3.5%) were excluded. For the reaction time analyses, we additionally excluded error trials (2.9%).

### Results and discussion

In Experiment 2, we also found a strong main effect of alerting (*β* = 0.039, *t* = 9.513, *p* < .001), showing shorter reaction times for alert trials compared with no cue trials. On average participants needed 775 ms (*SD* = 334 ms) in trials without a cue and 757 ms (*SD* = 333 ms) in trials with a cue. Using the same alerting effects of 10, 15, and 20 ms, the power simulations resulted in a power of 63% [52.76, 72.44], 97% [91.48, 99.38], and 100% [96.38, 100], respectively. We also found a main effect of set size (*β* = 0.255, *t* = 87.516, *p* < .001), showing shorter reaction times for target displays with fewer distractors. Each additional distractor increased the reaction times by about 31 ms (see Fig. 3), also resembling the hallmark of a set size effect in conjunction search tasks (Treisman & Sato, 1990). Again, the interaction between cue and set size (*β* = −0.013, *t* = −3.213, *p* = .001) was significant.

The accuracy analyses revealed a main effect of set size (*β* = 0.646, *z* = 4.969, *p* < .001), showing fewer errors with more distractors, an interaction between reaction times and set size (*β* = −0.501, *z* = −3.426, *p* < .001), showing that the influence of reaction times on accuracy diminished with more distractors, and an interaction between reaction times and cue type (*β* = 0.609, *z* = 2.035, *p* = .042), showing that the variance in reaction times was smaller with a preceding alerting cue compared with the condition without a preceding alerting cue. However, the main effect of cue type (*β* = −0.626, *z* = −2.820, *p* = .005) showed that accuracy was higher with a preceding alerting cue, which also speaks against a speed–accuracy trade-off (see Fig. 4).

As in Experiment 1, we found that visual alerting sped up visual search performance. Here, the reaction time difference between no cue trials and alert trials was smaller but still significant. Thus, even when controlled for temporal expectation and with higher target and distractor discriminability (high saliency of targets), alerting seems to benefit visual search performance (see Fig. 5). We also found an interaction between cue and set size. This interaction, however, was mostly driven by the smaller alerting effects with two stimuli and 16 stimuli. As can be seen by Fig. 4., an interaction seems not to be visually present. It is likely that these differences occurred just by chance as no systematic influences across the set sizes were identified.

## General discussion

This study showed that visual alerting positively affected visual search performance by significantly reducing reaction times in two visual search tasks. In Experiment 1, reaction times were on average 35 ms shorter with a preceding alerting cue. This was the case even though participants had to locate and discriminate the target. We also found that alerting effects were present in all distractor conditions. In Experiment 2, we replicated the findings. Here, participants benefitted by about 18 ms from a preceding alerting cue and the alerting effects also occurred in all distractor conditions. Crucially, the benefits did not occur at a cost of more errors. Taken together, the present findings demonstrate that visual alerting expedited performance across all levels of complexity.

In both experiments, visual search performance declined with the number of distractors. This classic set size effect (Bravo & Nakayama, 1992; Palmer, 1994; Pashler, 1987; Treisman & Sato, 1990) was greater in Experiment 1, with a more difficult discriminability between the target and distractors. In Experiment 1, reaction times increased with the numbers of distractors, while in Experiment 2, this relationship was less linear. The alerting effect, however, was not greater for set sizes with fewer distractors. If any, in Experiment 1, the alerting effect was greater with more distractors. However, this was not the case for Experiment 2. This argues that phasic alertness does not interact with selective attention. It seems that alerting exerts its effects equally across all levels of visual search efficiency. That is, participants benefit from alerting cues in situations where the visual search process is easy (i.e., small set size) and in situations where the visual search process is more complex (i.e., large set size; Townsend, 1990).

Previously, it was found that search times were shorter with an alerting cue for complex search tasks but not for easier efficient search tasks (Asutay & Västfjäll, 2017). In the present study, we found reaction time benefits in both Experiment 1 (low saliency of targets) as well as Experiment 2 (high saliency of targets) and irrespective of the number of distractors. Thus, the present findings clearly demonstrate that phasic alertness indeed modulates visual search performance across displays with low-salient targets as well as high-salient targets. However, in our Experiment 1, the target features were not markedly distinct from the distractors. According to Wolfe’s and Horowitz’s (2017) classification, all primary guiding features (i.e., colour, size, motion, orientation) that determine the visual search efficiency were made similar between the distractors and the target. In Experiment 2, only the orientation of the target letter differed from the distractors, making both visual search tasks rather challenging. It may be that alerting has limited benefits in efficient search tasks, as all available resources are already devoted to the target. Consequently, visual search performance may have reached ceiling, leaving little room for further improvement.

Contrary to that, are findings showing benefits in simple detection search tasks, but not in more complex discrimination search tasks requiring multiple processing steps (Jankovic et al., 2022). The authors argued that the first subtask (i.e., target detection) was facilitated in the more complex visual search task, but due to the additional step (i.e., feature identification), benefits by the alerting cue were no longer present. In the present visual search tasks, participants were required to not only detect the target, but also identify its orientation and subsequently select the appropriate response. Nevertheless, participants benefitted from an alerting cue. This contradicts with the temporal-period account proposed by Jankovic et al. (2022), which suggests that alerting enhances processing for a limited time period. According to this hypothesis, alerting effects are assumed to dissipate very quickly, so that more complex tasks involving multiple processing steps, such as target detection and feature identification, do not benefit from alerting cues. Reaction time benefits should only be observed in simple search tasks involving a single processing step of target detection. Note, not all models on visual attention would assume distinct processing steps of target detection and feature identification within the visual search process and rather assume a unified mechanism (cf. theory of visual attention; Bundesen, 1990). The temporal-period account also conflicts with a study linking phasic alerting to a task derived from the Trail Making Test (Dietze et al., 2023). In this study, participants’ task was to move a computer mouse in a 2D space and click on a series of numbers in ascending order. Despite reaction times being even longer than those observed in the present visual search tasks, it was found that alerting sped up responding to the first target (i.e., the first number) but left subsequent actions unaffected, regardless of the time needed to perform the actions. This suggests that even actions requiring the execution of complex motor plans can benefit from alerting. If the temporal-period account were accurate, the benefits of alerting should diminish when more than one processing step is involved. Alternatively, alerting effects could be restricted to discrete episodes so that task-set reconfigurations for new actions shut off the alerting effects.

The arousal-biased competition account, which proposes that arousal amplifies the priority of salient objects (Mather & Sutherland, 2011; but see Ásgeirsson & Nieuwenhuis, 2017), may only partially account for the previous findings. While this theory suggests that alerting should consistently lead to faster reaction times with pop-out targets, an alternative perspective posits an inverted *U* relationship between arousal-induced effects and performance (cf. Yerkes & Dodson, 1908). According to this hypothesis, the greatest benefits on behaviour should occur at intermediate levels of arousal and the smallest effects at very low and high levels of arousal. Alerting effects are not expected to emerge at extremely low and high arousal levels, as the alerting cues may not be capable of sufficiently altering the arousal level, similar to floor and ceiling effects. Therefore, the advantage of alerting might be influenced by the task demands, which determine the baseline arousal level required for optimal performance. In the present study, the average arousal level might have been elevated compared with the arousal level induced by simpler tasks, resulting in smaller reaction time benefits from the alerting cues. However, in the efficient search task by Asutay and Västfjäll (2017) and the complex search task by Jankovic et al. (2022), the arousal level could have been either too low or too high for alerting effects to emerge. This could also explain why some participants in the present study displayed large negative alerting effects, as the additional arousal boost by the alerting cue should only facilitate behaviour up to intermediate levels of arousal. The optimal level of arousal seems to vary from person to person and should depend on the nature of the task (see discussion on inter- and intraindividual variability by Dietze et al., 2023).

A major determinant of phasic alertness is the CTOA. In Experiment 1, the CTOA was the same across all trials, so that temporal expectation should have contributed to the alerting effects (Correa et al., 2004, 2005; Coull & Nobre, 1998). In Experiment 2, we controlled for temporal expectation by drawing CTOAs from a geometric distribution (Petersen et al., 2017; Poth et al., 2014; Weinbach & Henik, 2012). However, recent findings challenge the dissociation of alerting and temporal expectancy by means of nonaging probability distributions (Grabenhorst et al., 2019). In a psychophysical alerting study it was found that reaction times were shorter with longer CTOAs, which is indicative of temporal expectation influences (Dietze & Poth, 2023). Using nonaging probability distributions instead of aging or accelerated-aging distributions only reduces its impact on alerting (Lu et al., 2014). After all, it is likely that alerting effects will always reflect some sort of preparation. In the present study, it is not possible to determine whether the differences in alerting effects between Experiment 1 and Experiment 2 are primarily attributed to the changes in CTOAs or the variations in the difficulty of the search tasks. Nevertheless, the present findings demonstrate that alerting processes in combination with temporal expectation processes facilitate visual search performance. The benefits induced by the alerting cue were greater when temporal expectation was high (Experiment 1) and smaller but still existent when temporal expectation was low (Experiment 2).

In summary, the present findings revealed that visual alerting improves visual search performance. Reaction times were shorter with a preceding alerting cue for displays with low-salient targets as well as for displays with high-salient targets. In the present visual search task, participants had to locate and discriminate the target stimulus across several distractor conditions, highlighting that alerting is efficient for all types of complexity.

## Data Availability

The data and analysis code are available on the Open Science Framework (https://osf.io/w24zd/).
